# Evaluation of copy number variant detection from panel‐based next‐generation sequencing data

**DOI:** 10.1002/mgg3.513

**Published:** 2018-11-22

**Authors:** Ruen Yao, Tingting Yu, Yanrong Qing, Jian Wang, Yiping Shen

**Affiliations:** ^1^ Department of Medical Genetics and Molecular Diagnostic Laboratory, Shanghai Children's Medical Center Shanghai Jiao Tong University School of Medicine Shanghai China; ^2^ Institute for Pediatric Translational Medicine, Shanghai Children’s Medical Center Shanghai Jiaotong University School of Medicine Shanghai China; ^3^ Division of Genetics and Genomics Boston Children's Hospital, Harvard Medical School Boston Massachusetts

**Keywords:** chromosomal microarray analysis, copy number variation, next‐generation sequencing

## Abstract

**Background:**

Targeted gene capture and next‐generation sequencing (NGS) has been widely utilized as a robust and cost‐effective approach for detecting small variants among a group of disease genes. Copy number variations (CNV) can also be inferred from the read‐depth information but the accuracy of CNVs called from panel‐based NGS data has not been well evaluated.

**Methods:**

Sequencing data were acquired from patients underwent routine clinical targeted panel sequencing testing. Pathogenic CNVs detected from targeted panel sequencing data were evaluated using CNVs generated by two clinical accepted platforms, namely chromosome microarray analysis (CMA) and multiple ligation‐dependent probe amplification (MLPA) as benchmarks. CNVkit was used in our study to call CNVs from sequencing data using read‐depth information. CMA and MLPA tests were used to confirm and further assess the size and breakpoints of CNVs.

**Results:**

The size of CNVs detected using panel‐based NGS data are over 300 kb. The sizes of CNVs detected are slightly larger (102.3% on average) using the NGS platform than using the CMA platform, and the size accuracy improved as the size of variants increases. The breakpoints of CNVs detected using NGS data are quite close (within 2.3% of margin) to the breakpoints detected by CMA. CNVs on sex chromosomes, however, are less concordant between NGS and CMA platforms.

**Conclusion:**

Copy number variations covering adequate exons on autosomes can be accurately detected using targeted panel sequencing data as using CMA. CNVs detected from sex chromosomes need further evaluation and validation. Except for exon‐level deletion/duplication and CNV on sex chromosome, our data support the use of panel‐based NGS data for routine clinical detection of pathogenic CNVs.

## 1 INTRODUCTION

Copy number variants (CNVs) contribute to a large fraction of human genetic variation and have been known to play important roles in human diseases and evolution (Lupski, 2015). Both genomic disorders and many *Mendelian* diseases are caused by CNVs (Stankiewicz & Lupski, 2010; Zhang, Gu, Hurles, & Lupski, 2009).Chromosomal microarray analysis (CMA) is a powerful tool for detecting genome‐wide CNVs and had been recommended as a first‐tier diagnostic tool for patients with developmental delay of unknown etiology, autism spectrum disorders, and multiple malformations (Miller et al., 2010). Pathogenic CNVs were detected in 17% of these patients using chromosomal microarray testing (Yamamoto et al., 2014).Currently, this method is recognized as a “gold standard” method for CNV detection.

Next‐generation sequencing (NGS) based tests have rapidly become a routine clinical diagnostic tools for patients with suspected genetic disorders. Both panel and whole‐exome sequencing (WES) are used as effective assays for single‐nucleotide variations (SNVs) and small insertions/deletions (indels). Efforts are being made to detect CNV using WES data and the results further support the use of exome‐first approach for diagnostic purposes (Fromer et al., 2012; Ligt et al., 2013). While WES remains an effective but expansive test, well‐designed panel‐based NGS tests, a cost‐effective alternative, have been widely used for genetic diagnoses (Vrijenhoek et al., 2015). CNVkit is a software package calculating copy number ratio and discrete copy number segments based on on‐target reads and the nonspecifically captured off‐target reads (Talevich, Shain, Botton, & Bastian, 2016). In this study, we set to assess the analytical validity of CNV detection using CNVkit based on limited sequencing data extracted from targeted panel. We compared the submicroscopic pathogenic CNVs with those validated with CMA and exon‐level deletion/duplications with those validated by multiple ligation‐dependent probe amplification (MLPA).

## 2 MATERIALS AND METHODS

### 2.1 Subjects

A total of 450 patients underwent genetic testing at the Department of Medical Genetics, Shanghai Children's Medical Center from October 2015 to September 2017 were recruited in this study. All patients were prescribed with the panel‐based sequencing test due to possible diagnosis of genetic disorders by physicians from different department. Patients were informed of the risks and benefits and provided written informed consent for targeted panel sequencing.

### 2.2 Targeted capture and next‐generation sequencing

Patients’ genomic DNA was isolated from 2‐ml peripheral blood samples using a QIAamp Blood DNA Mini Kit® (Qiagen GmbH, Hilden, Germany). Three micrograms of DNA was processed through shearing using a Covarias® M220 Ultrasonicator system (Covaris, Inc. Woburn, MA, USA) to pieces of 150–200 bp in size. An adapter‐ligated library was produced with Agilent SureSelect Target Enrichment System (Agilent Technologies, Inc., Santa Clara, CA, USA) according to the manufacturer's instructions. The capture library was performed using an XT Inherited Disease Panel (cat No. 5190–7519, Agilent technologies, Inc.), containing 2,742 genes known to cause inherited disorders, covering only 10.5 Mb. Clusters were then generated by isothermal bridge amplification using an Illumina cBot station, and sequencing was performed on an Illumina HiSeq 2500 System (Illumina, Inc., San Diego, CA, USA). The raw data (fastq file) for each patient were obtained for CNV identification. The average sequencing depth of data used was 122, and more that 95.6% of targeted region was covered with 20 reads.

### 2.3 CNV identification based on read‐depth information

Copy number variations were identified using open source software called CNVkit (Talevich et al., 2016), a tool kit to infer and visualize copy number from targeted DNA sequencing data. Burrows Wheeler Alignment tool v0.2.10 (Li & Durbin, 2009) was employed for the alignment of sequencing data to the Human Reference Genome (NCBI build 37, hg 19) to generate bam files as input. Normal reference used for CNV identification were constructed using sequencing data from 10 normal males and 10 females which have previously validated without pathogenic CNVs by CMA. Default CNVkit settings were used for CNV identification individually.

### 2.4 Multiple ligation‐dependent probe amplification (MLPA)

Multiple ligation‐dependent probe amplification was used to detect exon‐level aberrations in six patients with clinical diagnosis of Duchenne muscular dystrophy (DMD). MLPA analysis was performed using the P034 DMD mix 1 and P035 DMD mix 2 kit (CE‐IVD; MRC‐Holland, the Netherlands) following manufacturer's instruction. Data were visualized and analyzed with Coffalyser software (MRC‐Holland, the Netherlands).

### 2.5 Chromosomal microarray analysis (CMA)

Genomic DNA was screened for CNVs using Agilent 4 × 180 K comparative genomic hybridization array (cat.No G4449A, Agilent technologies, Inc.) with an overall median probe spacing of 13 kb. Labeling and hybridization were performed following standard protocols. Microarray slides were scanned on a G2600D DNA Microarray Scanner (Agilent Technologies) using Agilent Scan Control with the preset Agilent scan profile (resolution 3 μm), then processed by the Agilent Feature Extraction software using the default protocol. The derivative log ratio spread (DLRS) was used for quality control. All arrays had a DLRS ≤0.2 and passed all quality control metrics (background noise <20, SNP call rate >0.6, restriction control >0.8 and reference correct >0.8). Data were visualized and analyzed with Agilent CytoGenomics software.

## 3 RESULTS

### 3.1 CNV identified by CNVkit

All CNVs identified by CNVkit were classified according to the ACMG guideline for CNV interpretation and reporting (Kearney, Thorland, Brown, Quintero‐Rivera, & South, 2011). A total of 61 pathogenic CNVs were detected in 53 patients, including 14 aneuploidy, 31 microdeletion/microduplication, one exon‐level deletion and seven complex cases. Sizes of these variants (aneuploidy cases not included) inferred by read‐depth information ranged from 304 to 76,404 kb.

### 3.2 CNV detection accuracy validated by CMA

**Figure 1:**
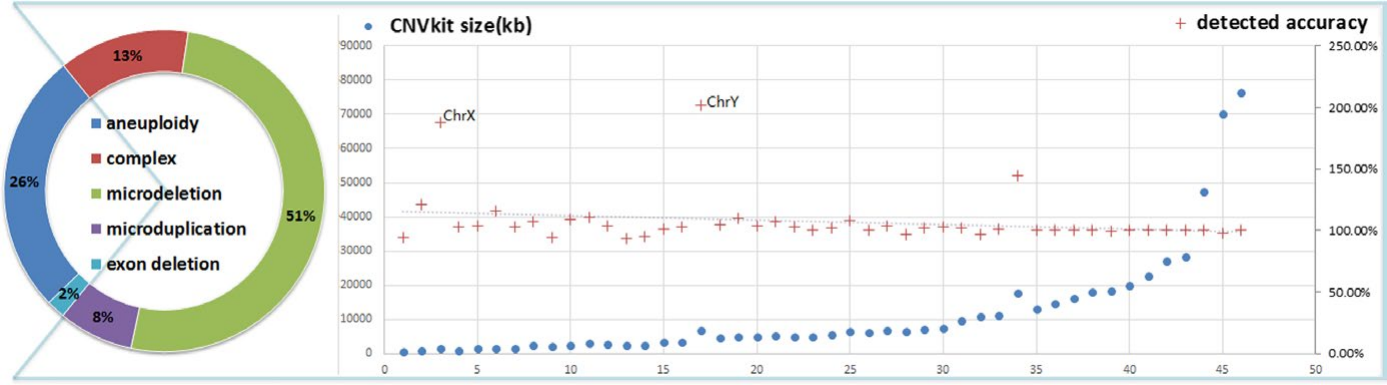
A doughnut chart of copy number variations (CNV) identified with CNVkit in patients. Among them, 46 submicroscopic variants are listed according to their size from chromosome microarray analysis detection. Accuracy of CNVkit detection improves as the size of CNVs grows

A total of 46 submicroscopic variants (568–76,404 kb) are validated with CMA. The size accuracy of CNV detected by CNVkit is evaluated against the sizes of variants detected by CMA (minimal interval referred by array probes) which was considered as standard. As a result, sizes inferred by CNVkit are slightly larger (average around 102.3% compared the size inferred by CMA platform) but the concordance improves as the size of the variants increases. Only two variants on sex chromosome have significantly discordant size call (Figure 1).

**Figure 2:**
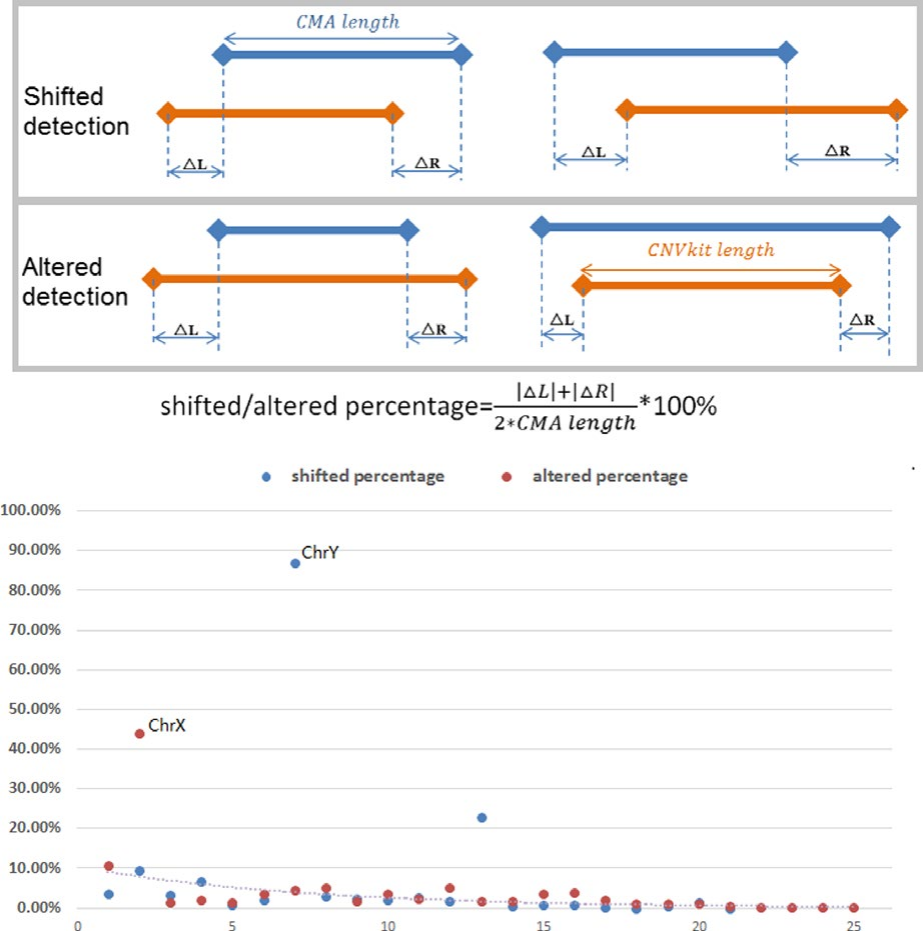
A schematic diagram of variants category for breakpoint estimation evaluation. Twenty‐one shifted and 25 altered variants are listed by size. The blue and orange segments represent sized detected from the chromosome microarray analysis platform and next‐generation sequencing platform using CNVkit

The proximal locations of CNV breakpoints are also evaluated. We classified the breakpoint differences into two types: shifted or altered, as illustrated in Figure 2. Percentage of shifting and altering are calculated based on detail coordinates generated by respective methods. Most variants are well located with altered or shifted percentage of margin <20% (average 2.3%), which suggests that the breakpoint concordance is quite good. Two variants with relatively larger altered or shifted percentage of margin (86.8% and 44.1%) were both on sex chromosome (Figure 2).

### 3.3 Exon‐level deletion validated by MLPA

CNVkit only detected one case with exon‐level deletion. To assess the CNV detection sensitivity using panel NGS data, we compared the CNVkit data with the MLPA data of six patients with clinical diagnosis of DMD. Four more deletion cases were diagnosed using MLPA but missed by CNVkit (Table 1). The exon deletion detected by both assay covers 304 kb and 10 exons and leads to out of frame deletion of the DMD gene.

**Table 1:** Five Duchenne muscular dystrophy (DMD) patients with their multiple ligation‐dependent probe amplification (MLPA) result detected by MLPA and CNVkit

Sample No.	DMD gene CNVs validated by MLPA			
	CNV detection from sequencing data	size	MLPA results	Reading frame check
1790	(−)	(−)	Exon 45–47 del	IN‐FRAME
1894	(−)	(−)	Exon 46–47 del	OUT‐OF‐FRAME
2994	(−)	(−)	Exon 8–9 del	OUT‐OF‐FRAME
3525	chrX:31645764–31950390 (deletion)	304 kb	Exon 46–55 del	OUT‐OF‐FRAME
4068	(−)	(‐)	Exon 48 del	IN‐FRAME

### 3.4 Complex CNV cases

**Figure 3:**
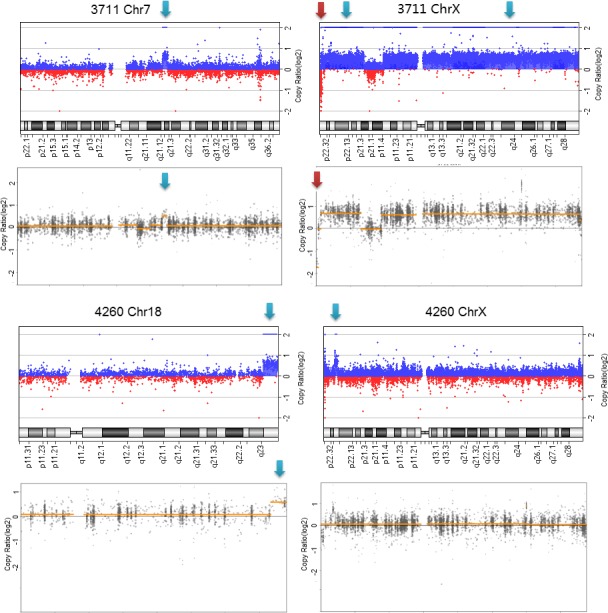
Complex variants in patients 3,711 & 4,260. The upper part is the detection of chromosome microarray analysis and the lower part is the detection of CNVkit. Red and blue arrows indicate deletions and duplications detected by each assay

Seven cases were detected with complex chromosomal rearrangements, with at least two segmental deletions or duplications (Table2). CNVs in five of these cases are consistent between CMA and CNVkit detection. Duplications on chromosome X in patient 3,711 were detected but unreported by the CNVkit due to gender identification error. The 1,664 kb duplication in patient 4,260 on chromosome X was undetected by CNVkit (Figure 3).

**Table 2:** Copy number variations (CNV) detection details from chromosome microarray analysis (CMA) and CNVkit

Sample No.	Detail of detection in complex case with at least two CNVs									
	CMA detection with agilent CGH array					CNVkit detection based on target panel sequencing				
	Chromosome	Cytoband	Start	Stop	Size (kb)	Type	Chromosome	Start	Stop	Size (kb)
3337	chr9	q21.11–q34.3	69197688	141053507	71,856	Duplication	chr9	71039515	141152931	70,113
	chrX	q21.1–q28	78852401	155232835	76,380	Deletion	chrX	78856047	155260060	76,404
3466	chr2	q35–q36.1	217664367	224706831	7,042	Deletion	chr2	217546699	224729907	7,183
	chr12	p12.1	22270505	24842178	2,572	Deletion	chr12	22499160	24953191	2,454
3581	chr10	p15.3–15.2	100026	3232499	3,132	Deletion	chr10	60500	3227376	3,167
	chr16	q22.3–24.3	74040004	90155062	16,115	Duplication	chr16	74168821	90294253	16,125
3711	chr7	q21.12–q21.13	87341941	90601433	3,259	Duplication	chr7	87230055	90583530	3,353
	chrX	p22.33	60701	772988	712	Deletion	chrX	60500	1401044	1,341
	chrX	p22.33–p11.1	1382606	58544060	57,161	Duplication	Undetected			
	chrX	q11.1–q28	61781133	155236288	93,455	Duplication	Undetected			
4078	chr5	p15.33–p14.1	71913	27061526	26,990	Deletion	chr5	10500	27119540	27,109
	chr18	q22.1–q23	65053487	78012819	12,959	Duplication	chr18	65037582	78016748	12,979
4176	chr4	p16.3–p15.1	143143	28194565	28,051	Duplication	chr4	13322	28228906	28,216
	chr9	p24.3–p22.2	185668	18214112	18,028	Deletion	chr9	10500	18114756	18,104
4260	chr18	q23	73587513	78012819	4,425	Duplication	chr18	73389723	78016748	4,627
	chrX	p22.31	6451691	8115193	1,664	Duplication	Undetected			

Note

CNVs in five of these cases are detected with satisfying accuracy (shifted and altered percentage <1%). Three duplications on chromosome X were undetected (shaded rows).

## 4 DISCUSSION

Targeted panel sequencing is now a routine diagnostic assay for detecting small variants for genetically heterogeneous conditions like ataxias (Fogel et al., 2014) or cardiovascular disease (Seidelmann et al., 2017) as well as for all known genetic disorders using subexome sequencing. These targeted panels are providing reasonable diagnostic yields with cost‐effective benefit. Simultaneous detection of CNVs from targeted panel sequencing data can further improve the technical utility of such panel‐based approach. Due to the fragmented nature of targeted panel sequencing and exome sequencing data, the only reliable way to detect CNV is to utilize the read‐depth of targeted regions. Identification of CNV from WES data has been proven to increase in the diagnostic yield (Pfundt et al., 2017). In this study, we evaluated the CNV detection based on read‐depth information from targeted panel sequencing data. The target sequencing panel we used covers 2,742 genes (10.9 Mb covered) related with inherited diseases. It is unknown if sparsely targeted NGS data will allow for reliable detection of CNVs, particularly pathogenic CNVs using only read‐depth information and how accurate in terms of size and boundaries of CNVs detected by panel‐based NGS data in comparison with CNVs detected by the “gold standard” platform CMA.

Based on read‐depth information and standard classification protocol, 61 pathogenic variants were detected from 53 patients by CNVkit, including aneuploidy, microdeletion/duplication, exon‐level deletion, and complex structural variants. Among them, 46 submicroscopic CNV ranged from 568 kb to 76 Mb were further validated with CMA. Size detection accuracy of these variants was compare between two assays, and most (43/46) CNV detected by CNVkit were consistent with results obtained on the CMA platform with accuracy around 102.3%. The only two discordant results were deletion from chromosome X and Y, ranging from 1,340 to 6,714 kb detected by the NGS platform. A previous study has evaluated several computational tools for all CNVs detected, and showed clear variation in the size detected (Samarakoon et al., 2014). When focused on pathogenic CNVs, target panel sequencing data based CNV detection seems to have passable size estimation capacity on most cases.

Breakpoint estimation is also evaluated between two platforms in detail. Shifting or altering percentages calculated based on the genomic coordinate of CNV detection are used in our study to determine the precision of breakpoint estimation. Among all these 21 shifted and 25 altered variants, breakpoint estimation was acceptable for most variants (shifting or altering percentage around 2.3%). The two discordant CNV detections mentioned above located on X and Y chromosome (3,325 and 2,564 kb detected by the CMA platform) have got relatively poor breakpoint estimation as well. Complex cases with CNVs are usually caused by balanced translocation in one of the parents, resulting in usually two telemetric deletion or duplication segments. Special events like complicated rearrangements or chromothripsis could result in even more segmental aberrations which are even difficult to detect and interpret. In our study, complex structural variants on chromosome X in one patient misguided the sex identification of the software, therefore the duplication of X chromosome was recognized as normal but the deletion of the SHOX gene region carried by the patient was still detected. The quality of CNV detection from sequencing data is affected by several technical factors, including heterogeneous sequencing depth as well as the underlying genomic architecture of regions (Ligt et al., 2013), which are more obvious on sex chromosomes. The fact that Y chromosome is the most highly enriched of the human chromosomes for CNV in the general population (Redon et al., 2006) and existence of pseudo‐autosomal region on sex chromosome may both contribute to difficulty of CNV detection. Thus CNV detection on autosome based sequencing data is more convincible than variants on sex chromosome for size accuracy and breakpoint estimation. Careful evaluation and additional validation is highly recommended for CNVs detected on sex chromosome.

For evaluation of smaller CNVs like exon‐level aberrations, all six DMD patients were tested with both MLPA and targeted panel sequencing. Genetic testing for exon level CNVs on the *DMD* should always report the exact number of exons engaged, in order to make clear genetic diagnosis based on the read frame rule (Monaco, Bertelson, Liechti‐Gallati, Moser, & Kunkel, 1988). Only one 304 kb deletion of the *DMD* was detected by CNVkit based on target panel sequencing data, encompassing 10 exons, and the deletion exons were confirmed by MLPA. The rest four DMD deletion cases were undetected, ranging from one to three exons revealed by MLPA. Thus, targeted panel sequencing data based approach was validated for detection of CNVs covering a sufficient number of exons. False negative is inevitable with deletions covering inadequate exons or size by targeted panel sequencing data. Previous study has got the same conclusion for WES data based CNV identification (Krumm et al., 2012).

Aneuploidy cases were not confirmed or further investigated in our study. Detection of aneuploidy from sequencing data has been stated in prenatal testing as lower false positive rates and higher positive predictive values using cell‐free DNA (Bianchi et al., 2014). Targeted sequencing of single‐nucleotide polymorphisms also holds promise for accurate detection of fetal autosomal trisomies, sex chromosome aneuploidies, and triploidy (Nicolaides, Syngelaki, Gil, Atanasova, & Markova, 2013). In our study, aneuploidy constituted 26% of all pathogenic variants detected. Patients with aneuploidy may not manifest typical phenotypes but screening these cases with targeted panel data could provide solid genetic diagnostic clue for physicians.

In conclusion, target panel sequencing data based CNV identification can help to detect submicroscopic copy number aberrations on autosome with acceptable accuracy. Detection of CNVs on sex chromosomes always needs confirmation using chromosomal microarray analysis. Smaller CNVs cannot be detected unless enough genomic region or exon numbers are covered. MLPA is still the best solution for targeted exon deletion/duplication testing with specific clinical indication like DMD. Detect pathogenic CNV from clinical exome sequencing data will increase the diagnostic yield of this assay with only computational effort, but false negative event should always be considered. CNV including some complex rearrangement can be accurately detected using panel NGS data, CNV detection should be recommended as part of the technical utility as for detecting SNV or del/dup for subexome (medical exome) panel testing.

## CONFLICT OF INTEREST

The authors declare that they have no competing interests.
